# Supplementary material for: Predicting the final grade using a machine learning regression model: insights from fifty percent of total course grades in CS1 courses
Source: PeerJ Comput Sci. 2023 Dec 11;9:e1689. doi: 10.7717/peerj-cs.1689 (PMC10773829; doi:10.7717/peerj-cs.1689)
Supplement: Supplemental Information 3 [file peerj-cs-09-1689-s003.pdf]

Universidad del Valle

CONSENTIMIENTO INFORMADO

NOMBRE DEL PROYECTO

CÓDIGO DEL PARTICIPANTE _____, (*Este mismo código aparece en el consentimiento que autoriza la participación)*

Lo invitamos a participar en una investigación para ___OBJETIVO___, con usted participarán __No__ estudiantes; lo que haremos es __ DESCRIBIR EN QUE CONSISTE LA PARTICIPACIÓN LISTANDO CLARAMENTE TODOS PROCEDIMIENTOS DEL ESTUDIO __, todo se hará en _____horas, como su participación es voluntaria debe realizar todas las actividades de lo contrario se retirará del estudio, en caso que no desees continuar puedes decir que no quieres y retirarse sin ningún problema ni explicación.

Garantizamos que su nombre ni datos personales serán mostrados ya que de aquí en adelante solo se identificará con el código asignado en la parte superior.

Es posible que __MENCIONAR LOS RIESGOS__, nosotros para evitarlo ____ contamos con el personal _DESCRIPCIÓN SEGÚN EL ESTUDIO__ adecuado para solucionarlo.

Los beneficios que obtendrás serán _____________, debe saber que no incurrirá en ningún gasto.

Acepto participar Si__ No___

Le daremos una copia de este documento.

Nombre del participante:
CC:

Nombre de quien presentó el consentimiento informado:
CC:

Se firma en Cali, a los ___ del mes de _______ del 202_.
